# Role of polyetheretherketone (PEEK) in arthroplasty and orthopaedics: a review of biomechanical properties, surface modifications, and clinical outcomes

**DOI:** 10.1007/s00590-025-04630-9

**Published:** 2026-01-07

**Authors:** Raju Vaishya, Abhishek Vaish, Akash Dubey, Karthik Vishwanathan, Abid Haleem, Mohd Javaid, Filippo Migliorini

**Affiliations:** 1 Department of Orthopaedics and Joint Replacement Surgery, Indraprastha Apollo Hospitals, New Delhi, India; 2 Department of Orthopaedics, Smt B. K. Shah Medical Institute & Research Centre, Sumandeep Vidyapeeth Deemed to be University, Vadodara, India; 3 Department of Mechanical Engineering, Jamia Millia Islamia, New Delhi, India; 4 Department of Trauma and Reconstructive Surgery, Martin Luther University Halle-Wittenberg, Halle, Germany; 5 Department of Orthopaedic and Trauma Surgery, Academic Hospital of Bolzano (SABES-ASDAA), Bolzano, Italy; 6 Department of Life Sciences, Health, and Health Professions, Link Campus University, Rome, Italy

**Keywords:** Biomaterials, Radiolucent implants, Surface modification, Stress shielding, Osteointegration, Joint replacement, Long-term outcomes

## Abstract

**Background:**

Polyetheretherketone (PEEK) has gained attention as an alternative to metallic implants in orthopaedic applications, including arthroplasty, due to its mechanical properties and biocompatibility. This review evaluates the role of PEEK in arthroplasty and other orthopaedic applications by analysing its biomechanical characteristics, surface modifications, and clinical outcomes

**Methods:**

A comprehensive literature review was conducted using PubMed, PubMed Central (PMC), Scopus, and Embase. Eligible studies included randomised controlled trials, cohort studies, preclinical research, and systematic reviews.

**Results:**

PEEK’s elastic modulus (3–4 GPa) closely matches that of human cortical bone, potentially reducing stress shielding and enhancing osseointegration. Its inherent radiolucency improves imaging capabilities, while various surface modification techniques, including hydroxyapatite coatings and nanopatterning, have been developed to enhance bone integration. Despite positive preclinical outcomes, clinical studies are predominantly focused on short- to mid-term results, with limited long-term data on implant survivorship and complications.

**Conclusions:**

While PEEK shows promise in orthopaedic applications, further research is necessary to establish its clinical efficacy relative to traditional metals. Addressing the current gaps in long-term studies, regulatory challenges, and manufacturing processes will be crucial for the broader adoption of PEEK implants in orthopaedic surgery. This review highlights the ongoing evolution of PEEK in the field and suggests directions for future research.

## Introduction

Orthopaedic surgery, including Arthroplasty, has long relied on metallic implants, predominantly cobalt-chromium-molybdenum (Co-Cr-Mo) alloys, titanium alloys, and stainless steel, which have served as the cornerstone materials due to their excellent mechanical strength, corrosion resistance, and biocompatibility [1]. These metals exhibit high elastic moduli—typically in the range of 110–240 GPa—which substantially exceed the elastic modulus of native cortical bone (~ 7 to 20 GPa) [2]. While this mechanical robustness ensures implant durability, the mismatch in stiffness can lead to undesirable biomechanical effects such as stress shielding, where the surrounding bone is underloaded, leading to bone resorption, implant loosening, and ultimately failure. Additionally, metallic implants pose challenges including the potential for metal hypersensitivity reactions attributed to ion release (notably nickel, cobalt (Co), and chromium (Cr)), and suboptimal postoperative imaging due to radiopacity and artifact generation, which complicates assessment of implant positioning and osseointegration [3], response to these limitations, there has been increasing interest in alternative materials that better simulate the biomechanical environment of bone, while maintaining sufficient durability and biocompatibility [4].

Polyetheretherketone (PEEK), a high-performance thermoplastic polymer, has emerged over the past decade as a promising candidate in orthopaedic applications, including joint arthroplasty and fracture fixation. PEEK’s elastic modulus (approximately 3–4 GPa) more closely approximates that of human cortical bone, potentially mitigating stress shielding and helping to preserve periprosthetic bone stock [5, 6]. Furthermore, PEEK is inherently radiolucent, which allows superior visualisation of the bone-implant interface and cement mantle on radiographs and advanced imaging modalities like Computed Tomography (CT) and Magnetic Resonance Imaging (MRI), addressing an important limitation of conventional metal implants. Another appealing feature of PEEK is its high chemical resistance and biological inertness, translating to low allergenic potential and favourable biocompatibility. However, this same inertness also challenges osseointegration, as unmodified PEEK surfaces do not naturally support bone cell adhesion or growth [5–8]. This has driven the development of novel surface modification techniques—ranging from hydroxyapatite coatings and sulfonation, to nanopatterning and incorporation of antibacterial agents—to enhance osteo-conductivity and reduce infection risk [7]. Additionally, advanced composites such as carbon fibre reinforced PEEK (CFR-PEEK) further improve mechanical strength and wear resistance, expanding PEEK’s utility in load-bearing joints [8–10].

Despite these promising attributes, significant gaps remain in the clinical validation of PEEK-based arthroplasty implants. Most existing clinical studies are limited to short- and mid-term follow-up, with a relative paucity of long-term data on implant survivorship, mechanical durability, wear-related biological responses, and comparative effectiveness versus traditional metals, especially in hip arthroplasty and complex revision surgeries [11]. Furthermore, while surface modifications show encouraging preclinical results, large-scale human trials substantiating their efficacy in reducing complications such as infection and aseptic loosening are still lacking. Cost, regulatory hurdles, and manufacturing challenges also impact the widespread adoption of PEEK implants [12].

The review aims to comprehensively synthesise the latest scientific evidence concerning using PEEK and its composites in Arthroplasty. It will examine physical and biological properties, advances in surface engineering, preclinical and clinical outcomes, comparative analyses with metal implants, and highlight key limitations and future research directions. This review seeks to inform clinicians, researchers, and device developers about PEEK’s evolving role in orthopaedic implantology and guide future innovations for improved patient outcomes by consolidating current knowledge and identifying gaps.

## Methods

A comprehensive literature review was conducted on 12 August 2025 to evaluate the biomechanical, biological, and clinical performance of PEEK and its composites in orthopaedic surgery. The electronic databases searched included PubMed, PubMed Central (PMC), Embase, and Scopus. The search strategy combined free-text terms and controlled vocabulary (MeSH/Emtree), using Boolean operators to maximise sensitivity and specificity. The core search string applied to PubMed was: *“polyetheretherketone” OR “PEEK” OR “carbon-fibre-reinforced PEEK” OR “CFR-PEEK”) AND (“arthroplasty” OR “joint replacement” OR “total knee arthroplasty” OR “total hip arthroplasty” OR “fracture fixation” OR “osteotomy” OR “spinal implants” OR “biomaterials” OR “osseointegration” OR “surface modification”*. Equivalent syntax was adapted for other databases. The search included all available records from database inception to the search date and no filters for study design were applied initially. Reference lists of eligible articles and relevant reviews were manually screened to identify additional studies.

### Eligibility criteria

Studies were included if they met the following criteria:

- *Population*: Human subjects undergoing orthopaedic procedures (arthroplasty, fracture fixation, spinal surgery, osteotomy), or animal/in vitro models evaluating PEEK for orthopaedic use.
- *Intervention*: Implants or components made from PEEK or its composites, including CFR-PEEK, modified PEEK (e.g., sulfonated, coated, nano-textured), or PEEK-based biomaterials.
- *Comparator*: Metallic implants, ceramic implants, UHMWPE, or unmodified PEEK surfaces, when applicable.
- *Outcomes*: Biomechanical properties, wear behaviour, osseointegration, biological response, functional outcomes, complications, survivorship, or imaging characteristics.
- *Study design*: Randomised controlled trials, cohort studies, case–control studies, case series, systematic reviews, preclinical animal studies, and laboratory research evaluating PEEK in orthopaedic contexts.
- *Language*: English.

Eligibility was defined to ensure that the evidence included in the review was directly relevant to the orthopaedic use of polyetheretherketone. All studies that evaluated PEEK or its composites in arthroplasty, trauma, spinal surgery, osteotomy, or related biomechanical and biological contexts were considered for inclusion. Studies were excluded when their scope was unrelated to orthopaedic, trauma, or spinal applications, such as work focusing on dental prosthodontics unless it provided insight into mechanisms of osseointegration relevant to musculoskeletal implantation. Articles examining PEEK solely as an industrial or manufacturing material without clinical, biomechanical, or biological implications were not considered. Finite-element analyses lacking experimental validation or without clear preclinical or clinical applicability were also excluded. Additional exclusion criteria encompassed conference abstracts without sufficient available data, publications not written in English, and article types without original research content, including editorials, commentaries, and letters. Studies investigating only polymers other than PEEK were likewise excluded, as they did not contribute to the aims of the review.

### Selection and data extraction

Two reviewers independently screened titles and abstracts, followed by full-text assessment. Data extracted included: material properties (elastic modulus, fatigue resistance), biological responses (osseointegration metrics, cytokine profiles), clinical outcomes (functional scores, complications, survivorship), wear characteristics, and imaging findings. Given heterogeneity in study designs and outcome measures, a narrative synthesis was performed. Summary tables were generated to compare material properties, surface modification techniques, and clinical outcomes of PEEK implants across orthopaedic applications.

## Results

### PEEK as a biomaterial

PEEK is a high-performance polymer that belongs to the polyaryletherketone family. Its molecular structure, comprising oxygen bridges and phenylene rings, confers exceptional mechanical and chemical resistance. PEEK is desirable for orthopaedic and spinal implants due to its unique chemical composition, providing strength, resilience, and durability. PEEK is the ideal material for orthopaedic implants due to its mechanical strength, biocompatibility, and elastic modulus, which closely resemble those of human bone. It can be used to make joint replacement components, spinal fusion devices, and other products that offer the body stability and support [13, 14]. Due to its mechanical strength, sterility resistance, and biocompatibility, PEEK is frequently used for various medical devices’ housing and structural elements, including electronic gadgets, portable surgical tools, and monitoring equipment. PEEK’s mechanical qualities, similar to human bone in strength, durability, and fatigue resistance, allow for improved load-bearing capacity and a lower danger of stress shielding. The high-performance thermoplastic polymer PEEK is quickly gaining popularity for the production of critical medical equipment. PEEK plastic offers tremendous promise for improving patient outcomes and the overall efficacy of medical implants, thanks to its unmatched strength, stability, biocompatibility, and heat resistance [5, 15, 16]. Unlike traditional plastics, PEEK shows exceptional resistance to ethylene oxide, radiation exposure, and autoclaving, among other repeated sterilisation techniques. This enables thorough cleaning of PEEK medical parts and components for use in delicate hospital settings. PEEK has completely changed the field of spine surgery. PEEK cages and interbody devices have shown exceptional mechanical stability, radiolucency, and biocompatibility in spinal fusion surgeries. PEEK spinal implants have shown promising results in clinical trials, including increased patient satisfaction, decreased postoperative pain, and high fusion rates. Because PEEK’s radiolucency facilitates the visualisation of the surgical site and enables precise implant placement, its use in minimally invasive spinal surgery has also gained popularity [17, 18]. PEEK is an excellent option for various medical treatments. The bone-like modulus PEEK is a high-performance polymer that is more flexible than ceramic and metal, a crucial characteristic for an orthopaedic device. Since PEEK is as flexible as cortical bone, it moves, flexes, and supports weight similarly to bone. A bone-like modulus is very important because orthopaedic implants are made to replace the strength and range of motion of bone. PEEK is an obvious choice for orthopaedic treatments due to its material properties. Decades of usage have proven its safety and biocompatibility. In comparison to other biomaterials, it is more robust and long-lasting [19, 20]. PEEK, particularly when reinforced (e.g., CFR-PEEK), offers mechanical benefits including a bone-like elastic modulus, high fatigue resistance, and improved imaging due to radiolucency. Table 1 summarises key comparative features between PEEK (and composites) and Co-Cr, the traditional gold standard for arthroplasty components.

**Table 1:** Comparison of polyetheretherketone (PEEK) and cobalt-chromium (Co-Cr) arthroplasty bearing properties

Property	PEEK/CFR-PEEK	Co-Cr	Significance
Elastic modulus	3–4 GPa [5, 6]	200–240 GPa [5, 6]	PEEK’s modulus is closer to bone
Radio-opacity	Radio-translucent [5]	Radio-opaque [5]	Better imaging with PEEK
Wear debris bio-response	Generally milder [13]	Potential metallic ion release [13]	Lower inflammatory risk with PEEK
Biocompatibility	Bioinert, needs modification [5, 11]	Good, but risk of allergies [5, 11]	Surface modification is critical for PEEK
Fatigue resistance	Adequate, long-term data pending [13]	Excellent [13]	Durability, a consideration for PEEK
Stress shielding	Reduced [5]	Pronounced [5]	Less bone loss with PEEK
Allergy risk	Negligible [10, 11]	1–2% (Co, Ni, Cr) [10, 11]	Better for PEEK, especially in sensitive pts

### Laboratory and animal model evidence

PEEK’s native surface is biologically inert—poor for bone integration—but substantial improvement is achieved through various surface modifications (Table 2). Laboratory experiments and animal modelling (rat femoral defect, MC3T3-E1 cell culture) show that advanced coatings and nano-texturing dramatically increase bone cell attachment, proliferation, and mineralisation, as well as enhancing hydrophilicity and reducing fibrous tissue formation [8].

**Table 2:** Surface modifications to enhance polyetheretherketone (PEEK) osseointegration and antibacterial properties

Modification	Osteointegration benefit	Antibacterial mechanism
Hydroxyapatite Coating [21–23]	Bone cell growth/attachment [21–23]	Minimal [21–23]
Sulfonation/Surface Etching [21–23]	Increases hydrophilicity, roughness [21–23]	Not Available
Silver/Copper Nanoparticle [21–23]	Cell attachment, anti-biofilm [21–23]	Disrupts bacterial membranes [21–23]
TiO2/Photoactivation [21–23]	Hydrophilicity, strong osteoblast adhesion [21–23]	Not Available
Polydopamine-Manganese (PDA-Mn) [21–23]	Upregulates osteogenic genes; bone regeneration [21–23]	May resist biofilm [21–23]
Antimicrobial Peptides [21–23]	Bone response [21–23]	Direct bactericidal effect [21–23]
Plant Polyphenol Incorporation [21–23]	Osteogenic potential; cell proliferation [21–23]	Inhibits bacterial growth [21–23]

A 2023 study using hierarchical porous PEEK in rat femur models reported significantly improved bone-implant contact (BIC), bone volume/tissue volume (BV/TV), and bone mineral density (BMD) compared to smooth PEEK [24]. Nano-textured and surface-activated PEEK also demonstrated enhanced cell adhesion and reduced inflammatory encapsulation [18].

### Wear, immunobiological response, and bio tribology

PEEK-based components shed debris that generally elicits a milder macrophage and foreign body response than metallic or UHMWPE particles [13]. However, CFR-PEEK bearings may, in some settings, provoke a more pronounced cytokine response than pure PEEK, warranting further chronic studies [25].

### PEEK as biomaterial for implants

Medical implant infection risks can be considerably decreased using PEEK implants, the primary requirement in the current scenario. PEEK materials enhance implant stability and patient comfort by reducing the development of bacterial biofilms and encouraging osseointegration. PEEK is poised to become a significant factor in improving patient outcomes for implant procedures as the demand for safer and more efficient implant solutions grows. PEEK implants have shown great promise in clinical settings, particularly in orthopaedics, dentistry, and spinal surgery, for reducing the risk of infection, enhancing implant stability, and improving patient comfort. PEEK’s contribution to reducing infection risks and improving patient outcomes in healthcare is anticipated to increase as research advances [26–28].

PEEK is a promising option for Arthroplasty due to its efficacy as a weight-bearing component in other applications. The effectiveness of PEEK as a femoral component and a secure substitute for Co-Cr has been the subject of numerous investigations. PEEK offers radiolucent material properties that enable artefact-free imaging during and after implantation, as well as performance characteristics such as modulus and flexural strength comparable to those of cortical bone, helping to preserve bone density. It is a better option than conventional metal implants because of its intrinsic qualities, which include mechanical resemblance to bone, biocompatibility, and resistance to bacterial adherence [26, 29].

### Clinical outcomes

#### Arthroplasty

PEEK is a high-performance polymer explored as an alternative to traditional metal alloys and ceramics in Arthroplasty, including total knee arthroplasty (TKA) and total hip arthroplasty (THA). The primary advantage of PEEK in these applications is its modulus of elasticity, which is much closer to that of natural bone than metallic implants. This property helps to minimise stress shielding, a common issue with stiffer metal implants that can lead to bone resorption and loosening. Additionally, PEEK’s radiolucency provides a significant benefit for postoperative imaging with CT and MRI, as it does not produce the dense artefacts associated with metal, allowing for a clearer view of the bone-implant interface.

##### Total knee arthroplasty (TKA)

In TKA, PEEK has been investigated for use in femoral components, with promising results in early wear simulation studies. A RCT compared posterior stabilized (PS) cemented PEEK TKR (PEEK femoral component, PEEK tibial component, polyethylene articulating liner and polyethylene patellar component) with posterior stabilized cemented Co-Cr-Mo TKR and reported no difference in the penetration of bone cement underneath the tibial plate, similar functional improvement (using the Knee Society Score), similar improvement in pain (using the VAS) and similar complications and survivorship of the prosthesis at 1 year postoperatively [30]. The distinctive radiolucency aids follow-up assessment (Table 3).

**Table 3:** Clinical outcomes in TKA: polyetheretherketone (PEEK) versus traditional materials

Outcome Parameter	PEEK implants	Traditional materials	Key clinical findings
Functional/Knee Society [30, 31]	Comparable [30, 31]	Comparable [30, 31]	No significant differences [30, 31]
VAS Pain Score [30, 31]	Comparable [30, 31]	Comparable [30, 31]	No significant differences [30, 31]
Complication Rate [30, 31]	Comparable [30, 31]	Comparable [30, 31]	No significant differences [30, 31]
Infection Rate [30, 31]	Similar or lower [30, 31]	Similar or higher [30, 31]	Favourable for PEEK [30, 31]
Cement Penetration [30, 31]	2.49 ± 0.61 mm [30, 31]	2.53 ± 0.68 mm [30, 31]	No significant difference [30, 31]
Survivorship (1 year) [30, 31]	Comparable [30, 31]	Comparable [30, 31]	Short-term parity [30, 31]
Imaging Quality [30, 31]	Superior (radiolucent, less artefact) [30, 31]	Inferior (artefacts) [30, 31]	Favours PEEK [30, 31]

A recent prospective study involving cemented implantation of PEEK femoral component, PEEK tibial component, polyethylene articulating liner, and polyethylene patellar component reported a gradual postoperative reduction in knee effusion, reduction in serum markers of inflammation, and significant improvement in functional outcome over 2 years. Functional assessment was performed using the Knee Society Score, and the volume of the knee effusion was evaluated using MRI [31]. 

##### Total hip arthroplasty (THA)

CR-PEEK has been used in acetabular cups for THA, offering a potential solution to reduce wear-particle-induced osteolysis. However, a significant challenge across both applications is the need for more long-term clinical data to assess PEEK’s wear resistance and overall longevity in vivo fully, as well as the high manufacturing cost of the material. Early to mid-term registry data suggest CFR-PEEK liners reduce stress shielding and enable thinner liners with similar wear rates, functional scores, and complication rates [32, 33]. A prospective case series investigating the use of CFR-PEEK as a liner for the acetabular cup reported four cases of aseptic loosening and subsequent need for revision THR, and the authors recommended against the use of CFR-PEEK [34]. 

#### Non-arthroplasty applications

##### High tibial osteotomy (plate fixation)

Lower hardware removal rates and safe healing have been reported for PEEK plates [35, 36]. A case series on medial opening wedge osteotomy using PEEK reported significant improvement in pain, activity levels, patient-reported functional outcomes, low revision rate to TKR, and low complication rate [37]. Another case series reported the feasibility and good outcome after HTO using PEEK material for a day care procedure [38]. Another comparative cohort study reported a lower rate of plate removal with HTO plates manufactured using PEEK material compared to the conventional metal HTO plates [36]. HTO plates made from PEEK material are also strong enough to permit earlier weight-bearing and accelerated rehabilitation postoperatively, facilitating early recovery of function [10, 39].

##### Cervical disc arthroplasty

PEEK cages demonstrated higher Neuro Disability Index (NDI) improvement, greater pain reduction, and lower hardware removal rates than metal disc prostheses at 12 months [40]. A prospective cohort study comparing cervical disc arthroplasty using PEEK-on-ceramic bearing and cervical discectomy and fusion for multi-level cervical disc pathology reported a lower rate of subsequent operative intervention for the cohort of patients treated with cervical disc arthroplasty [41]. The good results of cervical disc arthroplasty with PEEK-on-ceramic bearing continue to be maintained at long-term follow-up of 5 years postoperatively [42].

##### Proximal humerus plate

**Fig. 1:**
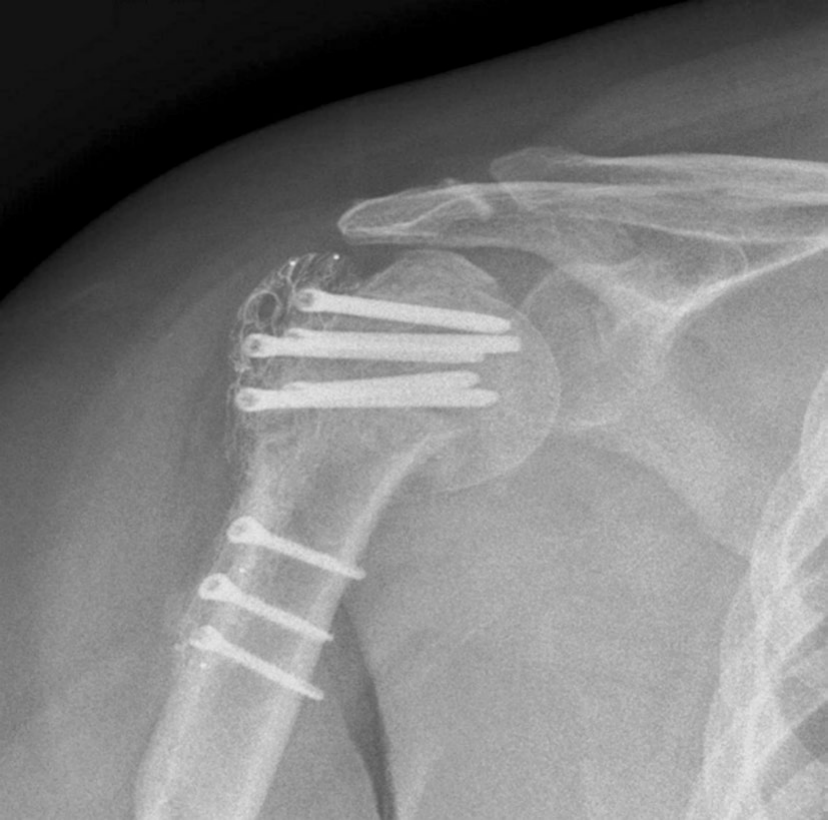
Postoperative radiograph of the right shoulder showing a radiolucent carbon-fiber–reinforced polyetheretherketone (CFR-PEEK) locking plate fixing a proximal humeral fracture (*Adapted from *[10]). (The radiolucent nature of the PEEK plate allows clear visualization of the underlying bone morphology, fracture line, and healing progression without the metallic artefacts commonly produced by titanium or stainless-steel implants. Screw positions, fracture alignment, and early callus formation can be assessed with improved clarity)

Proximal humerus plates made from CR-PEEK have been utilised as an alternative to the conventional titanium-made proximal humerus plates. A meta-analysis demonstrated that the CR-PEEK plate showed better Constant score, anterior elevation, lateral elevation, and adduction than the titanium plates, in treating proximal humeral fractures, and their complications were comparable [43]. A retrospective case series on 98 patients with proximal humerus fractures reported good functional outcome at a mean follow-up of 27.6 months and a minimum 12-month follow-up [44]. The study’s limitations were high loss to follow-up bias (34%), no statistical comparison of preoperative and postoperative functional scores, no comparison with the conventional material implant, and the possibility of selection bias due to the study’s retrospective nature (Fig. 1).

#### Imaging and allergy

PEEK materials’ radiolucency permits superior postoperative assessment of prosthesis position, bone density, and cement mantle integrity, with decreased metallic artefact in conventional radiographs and CT/MRI [45]. Furthermore, PEEK carries a negligible risk for allergy compared with metallic alloys, which can be significant in a subset of patients [10, 11].

## Discussion

**Fig. 2:**
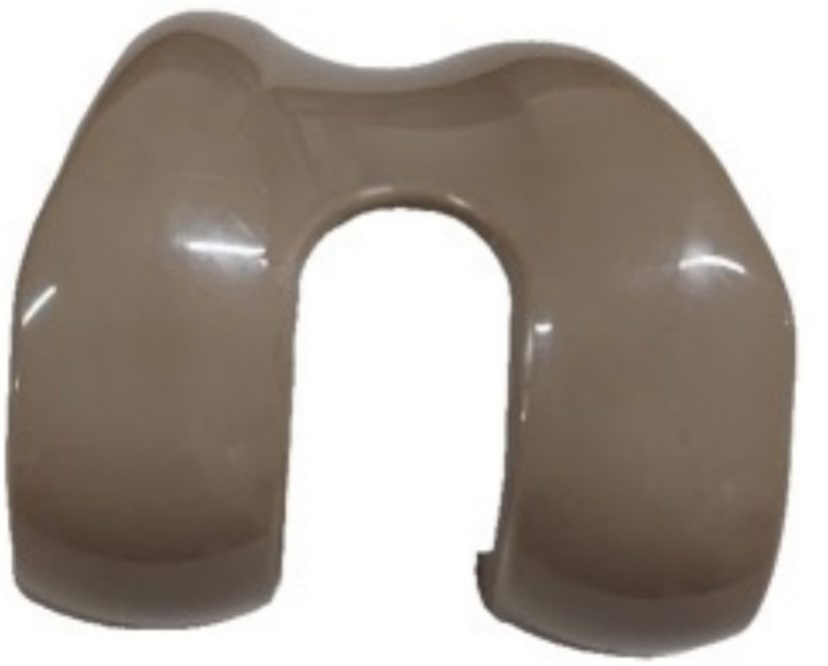
Polyetheretherketone (PEEK) femoral component of a Total Knee Arthroplasty (TKA), illustrating its anatomical contouring, smooth articulating surfaces, and design features comparable to conventional cobalt–chromium implants

This review demonstrates that PEEK has rapidly emerged as a viable alternative to traditional metallic materials in joint Arthroplasty and orthopedic trauma, owing to its unique mechanical, biological, and imaging benefits [5, 9]. The evidence across laboratory, animal, and clinical studies consistently indicates that PEEK-based implants deliver short- and mid-term clinical outcomes comparable to those of established metal implants [46]. This finding is particularly evident in TKA (Fig. 2), where RCTs and cohort studies reveal equivalent cement penetration, functional outcomes, pain relief, and complication rates when comparing PEEK to Co-Cr-Mo components [30].

**Fig. 3:**
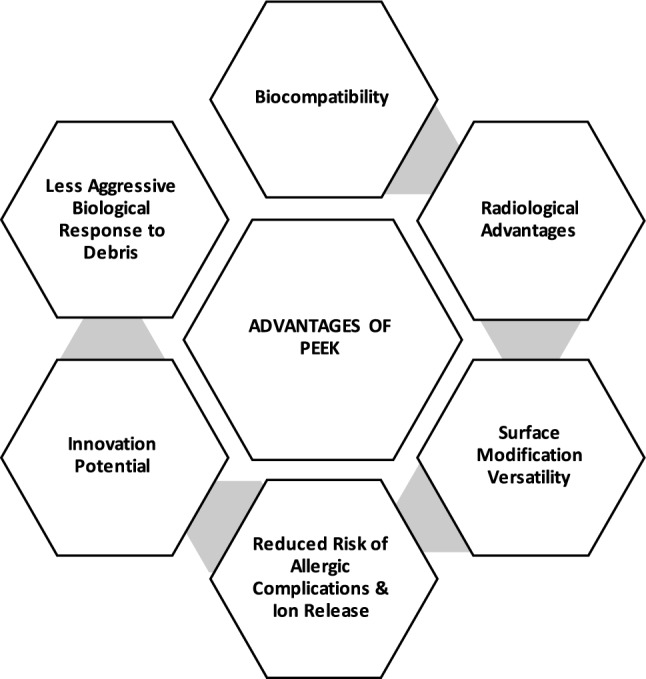
A schematic illustration of the key advantages of polyetheretherketone (PEEK) as a biomaterial, summarizing the biomechanical, biological, and functional advantages of PEEK relative to traditional orthopaedic implant materials

Notably, the elastic modulus of PEEK, which is significantly closer to that of natural cortical bone than metals such as Co-Cr or titanium, allows a more physiological load transfer (Fig. 3). This characteristic may play a crucial role in minimising the phenomenon of stress shielding and subsequent bone loss, a primary concern in traditional high-stiffness implants, especially for younger or osteoporotic patient populations [5, 47].

**Fig. 4:**
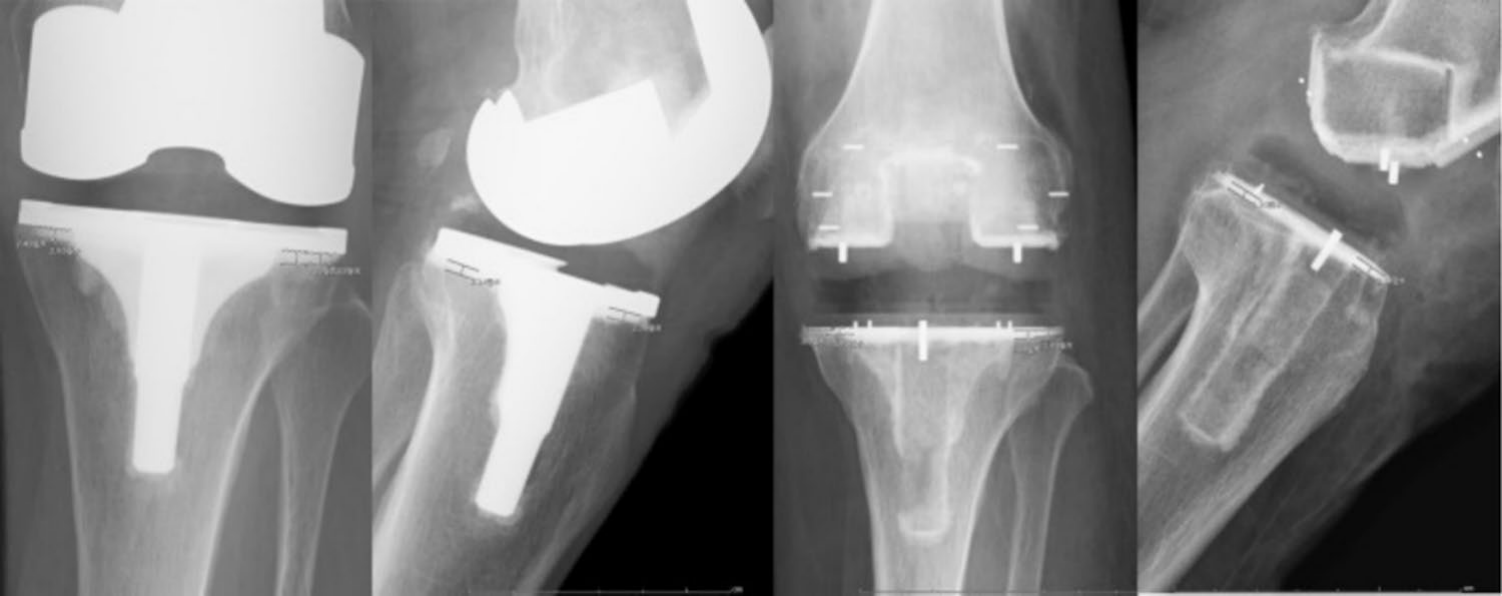
Comparative radiographic images after Total Knee Arthroplasty using conventional metal and PEEK implants, highlighting PEEK’s radiographic advantage in postoperative assessment, alignment verification, and early detection of loosening or osteolysis *(Adapted from *[30]*)*

Beyond its mechanical compatibility, PEEK’s radiolucency offers a substantial advantage for postoperative imaging (Fig. 4). Unlike metallic implants, PEEK does not obscure the bone-implant interface or generate artefacts on standard or advanced imaging modalities such as CT and MRI. This clarity facilitates early and accurate diagnosis of complications, assessment of implant integration, and monitoring of bone remodelling or osteolysis, outcomes which are otherwise compromised by metallic artefacts. In this respect, PEEK is particularly compelling for revision arthroplasty and in cases where precise serial imaging is essential [6, 48].

When directly comparing PEEK to other standard biomaterials—such as metals (Co-Cr, titanium alloys) and ultra-high molecular weight polyethylene (UHMWPE)—several distinctions emerge (Table 4). The modulus of PEEK lies between that of UHMWPE and metal but is markedly closer to bone than either, suggesting that PEEK has the potential for less stress-shielding and improved bone preservation [9, 49]. While traditional metals promote reliable osseointegration due to inherent bioactivity, and polyethylene offers good biocompatibility as a bearing surface, PEEK distinguishes itself by its negligible allergy risk, as it does not release sensitising ions such as nickel or cobalt [50]. This renders PEEK particularly attractive for patients with known or suspected hypersensitivity to metals.

**Table 4:** Polyetheretherketone (PEEK) versus other materials

Property	PEEK/CFR-PEEK	Co-Cr/Ti alloys	UHMWPE	Significance in arthroplasty
Elastic Modulus (GPa) [11, 51, 52]	3–4 (bone-like) [11, 51, 52]	110–240 (much higher) [11, 51, 52]	< 1 [11, 51, 52]	Lower stress shielding w/ PEEK [11, 51, 52]
Radiolucency [11, 51, 52]	Yes [11, 51, 52]	No [11, 51, 52]	Yes [11, 51, 52]	Superior imaging for PEEK/UHMWPE [11, 51, 52]
Osteointegration (native) [11, 51, 52]	Poor (bioinert) [11, 51, 52]	Good [11, 51, 52]	Minimal [11, 51, 52]	Needs surface modification [11, 51, 52]
Allergy Risk [11, 51, 52]	Negligible [11, 51, 52]	1–2% (Co/Ni/Cr) [11, 51, 52]	Rare [11, 51, 52]	PEEK favourable for allergies [11, 51, 52]
Fatigue Resistance [11, 51, 52]	High (short-midterm) [11, 51, 52]	Excellent [11, 51, 52]	Good [11, 51, 52]	Clinical data for PEEK growing [11, 51, 52]
Wear Debris Bioreactivity [11, 51, 52]	Mild [11, 51, 52]	Variable [11, 51, 52]	Inflammatory [11, 51, 52]	Favors PEEK for less osteolysis [11, 51, 52]
Cost & Manufacturing [11, 51, 52]	High, but declining [11, 51, 52]	Mature, cost effective [11, 51, 52]	Mature, cost effective [11, 51, 52]	Cost barrier being reduced [11, 51, 52]
Customizability/3D Printability [11, 51, 52]	High [11, 51, 52]	Moderate [11, 51, 52]	Low [11, 51, 52]	PEEK is suitable for novel designs [11, 51, 52]

PEEK, Polyetheretherketone; Co, Cobalt; Ni, Nickle; Cr, Chromium; UHMWPE, Ultra High Molecular Weight Polyethylene

An essential consideration for any implant biomaterial is its interaction with biological tissues and the body’s immune response to wear debris. The evidence suggests that PEEK and its wear particles provoke a less aggressive macrophage and inflammatory response than either metallic or polyethylene debris [53]. This property may be advantageous in reducing the risk of periprosthetic osteolysis over the long term. Nonetheless, it should be noted that CF-PEEK can induce a more pronounced cytokine response than pure PEEK in specific configurations, warranting further investigation regarding chronic wear and particle-induced biological reactions [54]. Despite these notable pros, PEEK is not without its limitations. The most significant challenge is its biological inertness—native PEEK integrates poorly with bone and may elicit fibrous encapsulation rather than true osseointegration. To address this, a range of surface modification strategies has been developed, including the application of bioactive coatings (e.g., hydroxyapatite, titanium dioxide, polydopamine-manganese composites), surface nano-texturing, and the incorporation of antibacterial or osteo-inductive agents [50]. Preclinical models and early clinical results indicate that these modifications can dramatically improve bone cell adhesion, proliferation, and mineralisation, upregulate osteogenic gene expression and enhance in vivo bone regeneration at the implant interface. Moreover, coatings with silver or antimicrobial peptides show potential for reducing bacterial colonisation, which may translate into a lower risk of periprosthetic infection [55], although this requires confirmation in large-scale human studies.

A pivotal issue for the field is that most clinical studies involving PEEK, especially in extensive joint replacements, are limited to short or mid-term follow-up—typically less than 5 years [30]. Robust, long-term registry data or RCTs extending beyond this timeframe are lacking, particularly for applications such as hip arthroplasty or other high-load scenarios. This gap constrains our ability to draw definitive conclusions about survivorship, late complications, fatigue failure, and rare adverse events over the lifespan of an implant. Mechanical data from laboratory settings are promising, indicating adequate fatigue resistance for physiological loads; however, newer forms of PEEK, especially highly porous or composite versions with advanced surface treatments, require more extensive biomechanical validation [33]. 

From a practical standpoint, adopting PEEK implants faces hurdles related to cost, regulatory approval, and potential surgical learning curves. While the manufacturing process for PEEK and advanced composites historically entailed higher costs than conventional metals or polyethylene, advances in additive manufacturing and broader market adoption are beginning to reduce these barriers [56]. PEEK’s amenability to 3D printing and patient-specific customisation may soon place it at the forefront of personalised implant design, especially for complex Arthroplasty, oncologic, or trauma reconstructions [10, 57, 58]. 

Another aspect deserving attention is the broader generalizability of current evidence. Many clinical trials and registry reports include highly selected patient cohorts—often excluding those with severe osteoporosis, immunosuppression, or higher functional demands. As such, caution is advised when extrapolating current findings to these populations. Additionally, most head-to-head randomised trials are concentrated on the knee, and evidence from other anatomical regions remains sparse.

PEEK and its composites offer a compelling biomaterial solution for arthroplasty and fracture fixation. Combining a bone-like elastic modulus, absence of metal ions, radiolucency, and a less aggressive immune response to wear particles underpins its unique profile. Distinct advantages are especially notable in patients at risk of metal allergy, requiring frequent imaging, or scenarios where bone preservation is paramount. The necessity of surface modification for reliable osseointegration is well-understood, and emerging technologies in coating and texturing have mitigated this concern to a significant extent.

PEEK is a durable, super-engineered polymer prized for its exceptional resistance to degradation, even in extreme conditions [10, 30]. Its advantages include high heat resistance, antiallergenic properties, and an elastic modulus similar to human bone, which makes it ideal for orthopaedic implants. PEEK also boasts excellent tensile strength, stiffness, and resistance to a wide range of chemicals and flame, emitting minimal smoke or fumes when burned. However, its high cost and complicated production process are significant drawbacks [28, 29]. The material’s high processing temperature and strength make manufacturing difficult and expensive, requiring specialised equipment. Additionally, PEEK’s low surface energy can hinder cell adhesion, posing a challenge for applications where biological integration is crucial (Table 5).

**Table 5:** Pros and cons of using PEEK as a biomaterial

PROS	CONS
General durability & resistance [10, 28–30]	Cost and Production Challenges [10, 28–30]
Incredibly durable for long-term use. [10, 28–30]	High cost compared to other technical polymers. [10, 28–30]
Exceptional resistance to degradation. [10, 28–30]	High processing temperature required. [10, 28–30]
Exceptional heat resistance. [10, 28–30]	Complicated production process requiring specialised tools/procedures. [10, 28–30]
Resistant to harmful substances, even under hot conditions. [10, 28–30]	Requires specialised methods and high-performance equipment. [10, 28–30]
Excellent resistance to a wide range of chemicals. [10, 28–30]	Challenging to cut and machine due to high strength. [10, 28–30]
[10, 28–30]	[10, 28–30]
Biocompatibility & Mechanical Properties [10, 28–30]	Performance & Biological Interaction Limitations [10, 28–30]
Antiallergic, reduces the possibility of allergic reactions. [10, 28–30]	Potential for deterioration between glass transition and melt transition temperatures (in high-temp applications). [10, 28–30]
Elastic modulus similar to human bone (ideal for orthopaedic implants). [10, 28–30]	Low surface energy, inhibits cell adhesion and integration (drawback in biological applications where cell integration is essential). [10, 28–30]
Exceptional tensile strength and impact resistance (even at high temperatures). [10, 28–30]	–
High hardness, outstanding tensile strength and stiffness (for high-load structural parts). [10, 28–30]	–
[10, 28–30]	[10, 28–30]
Safety Features [10, 28–30]	–

There are differences between first-generation PEEK and modern PEEK, mainly due to advances in formulation, processing, and applications over time. The first-generation PEEK, developed in the late 1970s and early 1980s, had the core polyether ether ketone polymer structure. Still, modern PEEK has evolved significantly with various grades and enhancements such as glass- and carbon-fibre reinforcements for increased strength and stiffness, which were not initially available. Additionally, modified versions now include additives like graphite, PTFE, and carbon fibre to improve wear resistance and friction properties, and there are high-temperature variants that maintain strength at higher temperatures than traditional PEEK. Tailored properties for specific fields, such as surgical implants, have also been developed, offering improved machinability, radiolucency, biocompatibility, and consistent tolerances compared to earlier types. While the fundamental chemical structure remains the same, modern PEEK benefits from decades of optimisation and diversification, making it a more refined and engineered polymer family with enhanced mechanical, thermal, chemical, and application-specific properties [7, 59, 60].

The future of PEEK in orthopaedic implantology is promising. This high-performance polymer has already been successfully modified for arthroscopic and joint replacement procedures and is expected to become a leading biomaterial for many other applications. Its unique mechanical properties, including an elastic modulus closer to bone than metal [17, 20], reduce stress shielding and improve load transfer, which enhances osseointegration and implant stability [61]. As researchers continue to find innovative ways to enhance PEEK’s functionality and biocompatibility, the material is poised to deliver impressive results in orthopaedics, dentistry, and spinal surgery.

Although the existing literature demonstrates that PEEK and its composites provide short- and mid-term outcomes comparable to traditional metallic implants, the evidence base remains limited by a pronounced scarcity of long-term clinical data. Most of the available studies report follow-up periods of less than 5 years, and only a small number extend beyond a decade, predominantly in highly selected cohorts. As a result, critical aspects of implant performance such as late aseptic loosening, long-term wear behaviour, fatigue failure under physiological loading, and rare adverse biological responses remain inadequately characterised. This gap is particularly evident in high-demand applications such as total hip arthroplasty, where only isolated series have raised concerns regarding long-term survivorship of CFR-PEEK liners, and where registry-level evidence is effectively absent. Moreover, the growing use of surface-modified and composite forms of PEEK introduces additional uncertainties, since most enhancements have demonstrated biological promise primarily in preclinical models, without robust human trials confirming their long-term clinical durability. These limitations constrain definitive conclusions regarding the life-cycle performance of PEEK implants and underscore the need for large, multicentre prospective studies and formal registry monitoring to clarify survivorship, late complications, and cost-effectiveness over the lifespan of modern orthopaedic implants.

Recent advances in surface engineering and materials science have further strengthened the clinical potential of PEEK implants. Modern techniques—including laser‑driven hydroxyapatite penetration, polydopamine-mediated nano-functionalisation, titanium-based nano‑coatings, hierarchical porosity, and antimicrobial nanocomposites such as silver nanoparticle (AgNP) coatings—have demonstrated substantial improvements in hydrophilicity, osteoblast adhesion, early osseointegration, and bacterial resistance. These contemporary strategies address the inherent bioinertness of native PEEK and are rapidly transitioning from laboratory studies to translational orthopaedic applications, offering enhanced integration and infection prevention in joint arthroplasty and trauma reconstruction [50, 62–66]. 

**Fig. 5:**
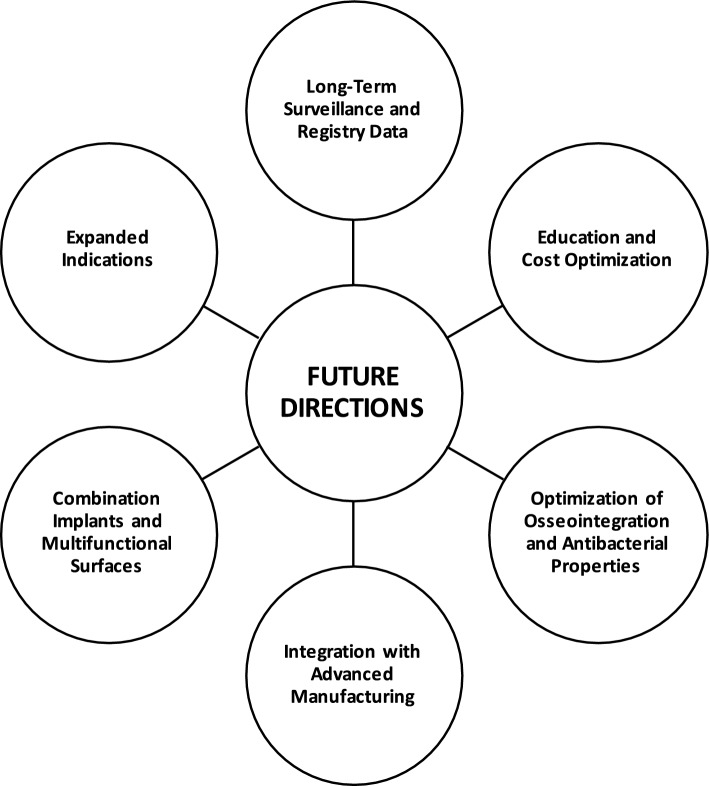
An infographic on the future directions in the applications of polyetheretherketone (PEEK) in orthopaedics, summarizing emerging innovations and research priorities for next-generation PEEK implants

Future research on PEEK in orthopaedics needs to focus on several key areas to maximise its potential. Long-term, multicenter clinical studies are essential to gather definitive data on survivorship, durability, cost-effectiveness, and rare complications. It’s also crucial to generate real-world registry data, develop multifunctional bioactive surfaces, and integrate the material more fully with 3D printing technologies. Prioritised studies should also assess wear patterns, biological responses to different PEEK composites, and the economic implications of routine PEEK implant use for optimising patient care and resource allocation (Fig. 5).

## Conclusion

PEEK offers biomechanical and imaging advantages that make it an attractive alternative to traditional metals in orthopaedic surgery, although current evidence is dominated by short- and mid-term data. The lack of robust long-term studies limits definitive conclusions regarding survivorship, late complications, and durability under physiological loading. Future research should prioritise large prospective cohorts, registry-based monitoring, and validation of surface-modified and composite formulations to clarify long-term performance and optimise clinical use.

## Data Availability

Data is provided within the manuscript or supplementary information files.
